# Deliberate Practice and Motor Learning Principles to Underpin the Design of Training Interventions for Improving Lifting Movement in the Occupational Sector: A Perspective and a Pilot Study on the Role of Augmented Feedback

**DOI:** 10.3389/fspor.2021.746142

**Published:** 2021-11-02

**Authors:** Luca Oppici, Kim Grütters, Alessandro Garofolini, Robert Rosenkranz, Susanne Narciss

**Affiliations:** ^1^Psychology of Learning and Instruction, Department of Psychology, School of Science, Technische Universität Dresden, Dresden, Germany; ^2^Centre for Tactile Internet With Human-in-the-Loop (CeTI), Technische Universität Dresden, Dresden, Germany; ^3^Institute for Health and Sport (IHES), Victoria University, Melbourne, VIC, Australia; ^4^Acoustic and Haptic Engineering, Faculty of Electrical and Computer Engineering, Technische Universität Dresden, Dresden, Germany

**Keywords:** back pain, low back, educational approach, expertise, skill acquisition, feedback modality, spine

## Abstract

Spine posture during repetitive lifting is one of the main risk factors for low-back injuries in the occupational sector. It is thus critical to design appropriate intervention strategies for training workers to improve their posture, reducing load on the spine during lifting. The main approach to train safe lifting to workers has been educational; however, systematic reviews and meta-analyses have shown that this approach does not improve lifting movement nor reduces the risk of low back injury. One of the main limitations of this approach lies in the amount, quality and context of practice of the lifting movement. In this article, first we argue for integrating psychologically-grounded perspectives of practice design in the development of training interventions for safe lifting. Principles from deliberate practice and motor learning are combined and integrated. Given the complexity of lifting, a training intervention should occur in the workplace and invite workers to repeatedly practice/perform the lifting movement with the clear goal of improving their lifting-related body posture. Augmented feedback has a central role in creating the suitable condition for achieving such intervention. Second, we focus on spine bending as risk factor and present a pilot study examining the benefits and boundary conditions of different feedback modalities for reducing bending during lifting. The results showed how feedback modalities meet differently key requirements of deliberate practice conditions, i.e., feedback has to be informative, individualized and actionable. Following the proposed approach, psychology will gain an active role in the development of training interventions, contributing to finding solutions for a reduction of risk factors for workers.

## Introduction

Low back injury and pain are conditions affecting a large portion of population worldwide, with deleterious psycho-social and economic consequences (Dagenais et al., 2008; Hoy et al., 2014). Different risk factors exist and are known to interact for low back pain: primarily biological, biomechanical, psychological, and social factors (Waddell, 1987; McGill, 2016). In the occupational sector, the type and quality of performed movement are considered major risk factors (Marras et al., 1993; Coenen et al., 2013). High frequency of lifting movement exposes workers in specific occupational domains, such as construction and material handling at a higher risk of injury (Pope et al., 2002; Coenen et al., 2014; Parreira et al., 2018). Stresses on the spine and consequently the injury risk factors vary according to certain aspects of the lifting movement. For example, spine flexion and torsion, lifting speed, and distance to the lifted object are critical movement features. Training/teaching workers to improve their movement and decrease spine load is a critical preventative measure for low back pain and injury in the occupational sector.

Historically, the main approach to teach and train workers safe lifting movement has been educational (we make a clear distinction between teaching and assisting strategies [e.g., exoskeletons], and here we consider the main adopted strategy for promoting movement learning); however, this approach has some limitations, and its effectiveness has been questioned. In educational interventions, notions on body anatomy and lifting biomechanics, how to maintain a correct lumbar posture (i.e., without flexion), and the optimal lifting technique are typically delivered using teaching material (e.g., slides and video) (Donchin et al., 1990; Daltroy et al., 1993, 1997; van Poppel et al., 1998). Importantly, limited time is dedicated to practicing, training, and acquiring a “safe” lifting movement. For example, a typical intervention would be comprised of two to four lessons of 90 min each, led by a physiotherapist in small working groups, whereby workers listen about and practice lifting postures (Donchin et al., 1990; Daltroy et al., 1993). Multiple systematic reviews and meta-analyses have shown that this approach does not reduce low back injuries and episodes of pain (Maher, 2000; van Poppel et al., 2004; Verbeek et al., 2011; Steffens et al., 2016; Sowah et al., 2018; Huang et al., 2020).

We contend that one of the main limitations of the educational approach lies in the amount, quality, and context of practice of the lifting movement. The amount of supervised practice is generally very low (~1–2 h), and this practice is dis-embedded from the workplace environment (i.e., practice occurs in locations other than the workplace). This likely reduces the potential effectiveness of such intervention on changing a worker's lifting behavior. The knowledge on lifting-related biomechanical and anatomy aspects taught in the classroom remains abstract and dis-embodied (i.e., it is not acquired and expressed through the body), and the dis-connection between practice context and the workplace limits transfer of the practiced lifting movement to daily contexts. These elements of the educational approach (training is dis-embodied and dis-embedded), however, should not come as a surprise, considering that the approach draws from the Back School (Forssell, 1980) and is not grounded in psychology and pedagogical principles. Occupational therapists typically design and deliver the intervention, and psychology (in its broad term) has often been overlooked. We are not arguing that providing workers with notions on biomechanics and anatomy, and opportunities to practice isolated lifting movement is pointless; rather it should represent a first step in a training program and should be combined with an embodied and embedded intervention as we describe in the following.

This article is structured in two main parts: a theoretical perspective and an empirical pilot study. In the first part, we argue for integrating psychologically-grounded perspectives of practice design in the development of training interventions for safe lifting. Research has shown that experts lift “better” than novices and are generally at a lower risk of injury: expertise modulates workers' movement and risk of injury (Marras and Karwowski, 2006; Plamondon et al., 2014; Gagnon et al., 2016). Lifting consistently and reliably with a safe lumbar spine posture should then be considered as an expert movement. Principles from expertise (deliberate practice and knowing in practice; Ericsson et al., 1993; Billett et al., 2018) and motor skill learning literature (augmented feedback; Magill and Anderson, 2012) should guide the design of appropriate training interventions. This article focuses on the role of augmented feedback for an embodied and embedded intervention in the workplace. These principles are discussed on a theoretical level and, while they can be used to target different movement-related risk factors, the argument gradually focuses on the risk factor we have decided to target: spine flexion. Flexion (or bending) of the spine during lifting is a relevant risk factor for low back injury (Fathallah et al., 1998; Taylor et al., 2014). In fact, repetitive and loaded (i.e., with an external load) flexion of the trunk can damage tissues of the spine (Callaghan and McGill, 2001; Adams and Dolan, 2012), and flexion of the lumbar area apply anterior share forces, further increasing the injury risk (Potvin et al., 1991; McGill et al., 2000). In the second part, we present a pilot study where we examined how tactile and auditory feedback modalities influenced the reduction of spine flexion during lifting. The results of this pilot provide insights on benefits and boundary conditions for the implementation of augmented feedback in the workplace. Importantly, acknowledging that risk factors for low back pain are multifactorial, the approach we discuss throughout the manuscript addresses the issue of training the lifting movement and reducing movement-related risk factors. It should not be seen as *the* one and only preventative intervention for low back pain.

## Deliberate Practice Framework and Principles of Motor Learning for Designing a Training Intervention: A Theoretical Perspective

### The Deliberate Practice Framework Applied to Training Lifting

The deliberate practice framework contends that expert performance is attained through deliberately engaging for thousands of hours in practice activities with the aim of optimizing performance (Ericsson et al., 1993; Ericsson, 2003). A key and defining feature of expert performers is their consistent and reliable achievement of successful performance in a variety of contexts and situations. The seminal work of Ericsson and colleagues has shown that the amount of deliberate, effortful, individualized, and guided practice with the aim of specifically improving certain aspects of performance is what distinguished expert, amateur musicians and music teachers, with experts engaging in deliberate practice up to three times more than their counterparts (Ericsson et al., 1993). While some issues have been raised (e.g., Macnamara et al., 2016; Macnamara and Maitra, 2019), these findings have been replicated in various domains, including art, music, medicine, and sport (for an overview see Baker and Farrow, 2015; Ericsson et al., 2018). Importantly, not all types of practice can classify as deliberate practice, and to be defined as such some key requirements have to be met: (i) individualized design of effective practice, (ii) active responses to a task with an explicit goal, immediate feedback, and repetitions, and (iii) individualized assessment of skill and design of future practice (Ericsson et al., 1993; Ericsson and Harwell, 2019; Ericsson, 2020).

Deliberate practice is particularly relevant for complex skills (e.g., decision making in chess; Chase and Simon, 1973) and, consequently, should guide the design of an intervention to train safe lifting, given its movement complexity. Lifting an object from the ground or a shelf, keeping a neutral, safe lumbar spine is quite a complex movement with multiple degrees of freedom to control (e.g., knee, hip, and intersegmental spine angles). In this movement, the body faces the challenge of coordinating the different parts of the body to move its center of mass vertically and horizontally to reach for and move an object, ensuring that the center of mass remains within the base of support (to avoid falling over), while keeping a reduced lumbar flexion angle. Ankles, knees, hips, and trunks have to be finely controlled and coordinated to achieve such a complex goal. For example, a flexion only of the hips will inevitably result in lumbar flexion (unless the person has extremely flexible hamstrings), or flexion only of the knees will result in the body center of mass falling outside the base of support, or flexion only of the knees and ankles will result in an unstable posture (base of support is reduced) and high stress of the knee joint. Considering this complexity, the development of an appropriate movement for safe lifting can only be achieved through a considerable amount of hours of practice.

An example that shows the importance of deliberate practice in training lifting comes from weightlifting. Typically, athletes in weightlifting disciplines have an average weekly lifting volume of 10 to 40+ tons (Bazyler et al., 2018; Travis et al., 2020). However, they have a relatively low rate of back injury, which is lower or does not differ from other sports with a much lower lifting volume (Aasa et al., 2017; Keogh and Winwood, 2017). Expert weightlifters have been shown to lift with more efficient movement dynamics than novices (Adelsberger and Tröster, 2013). Amongst other factors (e.g., healthy lifestyle and enhanced strength and flexibility), a high amount of effortful practice with targeted goals, instruction, and feedback (i.e., deliberate practice) surely play a critical role in allowing athletes to lift high volume with a relatively low rate of injury (Ronai, 2020). Weightlifters learn to lift safely first and only then they start increasing the lifted weight (Favre and Peterson, 2012). In the occupational context, most times the opposite happens: workers start lifting weight from day one and only at a later stage they may undertake a lifting training.

Research in motor skill learning indicates how well-designed practice promotes the development of movement forms that are functional to achieve the goal of a task (Magill and Anderson, 2017), corroborating the importance of deliberate practice for attaining an expert status. Through practice, individuals search and explore different perceptual-motor modalities to negotiate the task requirements, and stabilize the patterns that are goal-relevant (Fowler and Turvey, 1978; Newell, 1991; Pacheco et al., 2019). Learners engage in a non-linear transformational process, whereby their cognitive, perceptual, and motor systems tune to the main features of the task at hand and the environment in which the movement is performed (Chow et al., 2011; Gollhofer et al., 2013). Cognitive processes improve and contribute to the planning of a learner's intention to perform a certain movement (Bruineberg and Rietveld, 2014); perception tunes to the most reliable and “informative” information to guide action (Fajen, 2005; Warren, 2006); synergies form between different body components to allow flexible, yet stable control of the abundant degrees of freedom of movement (Asaka et al., 2008; Latash, 2010). These processes are not separate, but emerge from their confluence and shape each other throughout practice. As such, the observable movement emerges from the dynamic, non-linear, inter-play of these processes (not only one isolate process), and it is only through extensive practice of these dynamics that goal-relevant movement solutions are established, i.e., performance and learning improves. Applied to lifting, deliberate practice will enhance, just to name a few, an individual's ability to (i) plan and goal-orient their intentions to lift in a certain way (e.g., “I can try and flex my hips a bit more to improve my balance and reduce lumbar flexion”), (ii) perceive goal-relevant proprioceptive information about current position of and relation between body parts, (iii) coordinate synergistically ankles, knees, hips, and trunks, and most importantly (iv) integrate all these processes to achieve lifting with a reduced lumbar flexion.

It should be quite apparent at this point that the traditional educational approach, based on general lifting knowledge and little practice, presents limitations for training the lifting movement, and (we argue) should be integrated with the deliberate practice approach. Cognition is only one component of the dynamics of movement and learning process explained above. Knowing that you need to bend your leg and not your back when lifting, does not necessarily transfer into moving correctly. For example, nurses know how to (theoretically) perform safe lifting but they do not apply those principles into their movement (Kuipers et al., 2016), and telling individuals to lift with their leg and not their back is not as straightforward as it may sound (Beach et al., 2018). Knowledge acquired in the classroom about lifting biomechanics and suitable postures represents only the first step to get an idea on how a suitable lifting movement may look like and what aspects of a movement should be considered for developing a safe behavior. This knowledge and cognition need to be applied to and expressed through the lifting movement (i.e., embodiment) (Clark, 1999; Shapiro, 2019). In a continuous cyclical relation, knowledge shapes, and is also shaped by, the interweaving of perception, cognition and action during the repeated performance of lifting movement. Coupling knowledge with movement would allow people to feel their body when they move in different ways (e.g., bending or straightening their back) and perceive key information from their body that “tells” them when they are moving correctly. Workers will then “build” their *own* knowledge of their *own* safe movement.

### Deliberate Practice Applied to Training Lifting in the Workplace

While in some domains individuals have the opportunity to dedicate a considerable amount of time for deliberately practicing a skill in preparation for performance events (e.g., in sport and music), in the occupational domain workers do not have this “luxury” and deliberate practice has to be integrated in their daily performance of lifting activities. While it will pose some challenges because practice has to fit with work requirements, embedding practice directly in the workplace has many advantages. It will promote the development of lifting behavior through a continuous interaction between a worker and the characteristics of the work environment in which lifting takes place.

Research in occupational expertise has shown the importance of embedding deliberate practice into the workplace (for an overview see Billett et al., 2018). Knowledge and expertise are not only shaped by practice *per se*, but also by the contextual factors in which practice takes place. In fact, expertise is predicated on workers' ability to apply general (or canonical) knowledge to the challenges of the specific working contexts they are confronted with (Billett, 2001b). The ability to detect and exploit the action possibilities that the working environment offers, and to enact a suitable behavior is what distinguishes experts from novices (Billett, 2001c). For example, hairdressers enact different strategies and develop different expertise to successfully deal with their clients depending on the characteristics of the suburb they work in (e.g., high or low socio-economic areas) (Billett, 2001a). For these reasons, the importance of learning skills and developing expertise directly in the workplace is appreciated in a variety of working domains (Illeris, 2003; Fenwick, 2008).

Research in motor control and learning also supports practicing motor skills in the specific context in which the behavior is intended to apply (representative design, for an overview see Dhami et al., 2004). Movement is controlled on the information emerging from the interaction of a performer and the environment in which a task is performed (e.g., the informational ratio between object distance and braking capabilities in car braking; Fajen, 2005), and learning is specific to the information for action present during practice (specificity of practice hypothesis; Proteau, 1992). As such, practicing a skill directly in the performance environment (the workplace in occupational domain) or in a context that contains elements of it promotes the development of skills adequate to achieve the movement goal in such contextual factors. The elements present in practice shape how perception, cognition, and action emerge and develop. For example, perception develops differently depending on the contextual properties in which a motor skill (e.g., football passing) is practiced (Oppici et al., 2017), and generally motor skills learned in the laboratory transfer poorly to contexts outside the lab (i.e., the learned behavior is not functional to requirements outside the lab) (Barnett and Ceci, 2002).

The working context surely plays an important role in shaping how workers develop lifting behavior throughout their career, and this should be taken into account in the design of training interventions. The type of weight to lift (e.g., a small or large box), the location a weight is lifted from and placed onto, the environment surrounding the worker, and the interactions of all these elements with the psycho-physical characteristics of a worker will “dictate” how a worker approaches and performs the lifting task. Furthermore, these dynamics influence how a suitable, safe movement may look like (e.g., higher knee flexion in boxes with low handle). Research has shown heterogeneity in flexion-extension movements across healthy participants (Beaudette et al., 2019; Zwambag et al., 2019) and also that different lifting techniques are required to accomplish different lifting tasks in different contexts (van Dieën et al., 1999; Swinton et al., 2012). It follows then that *the* optimal lifting technique for all individuals (i.e., one size fits all) does not exist and cannot be taught in educational programs. Even ergonomists and safety professionals have slightly different ideas on how the optimal lifting technique looks like (Abdoli-Eramaki et al., 2019). This all means that every worker should be invited and put in the condition to explore and develop their own best movement pattern to lift safely, and this should occur in the context in which a worker will perform lifting on a daily basis (i.e., in their own workplace), not in a de-contextualized practice setting.

In summary, we argue that safe lifting should be trained applying the deliberate practice framework directly in the workplace. This may seem a trivial argument, considering that workers already practice a high volume of lifting in the workplace as part of their work routine (e.g., delivery workers lift thousands of boxes in a year), and that eventually, after several years of practice, workers will improve their lifting behavior (expert workers typically lift with a smaller lumbar spine flexion than novices; Plamondon et al., 2010, 2014; Boocock et al., 2015; Riley et al., 2015; Gagnon et al., 2016). However, it is known that mere practice of lifting is not sufficient to improve the movement and attain an expertise level (Gagnon, 2003, 2005), and the category at a higher risk of injury is the novice and young population at an early stage of their career. In turn, deliberate practice is much more than “simply” performing lifting on a daily basis, and this approach aims at fast-tracking the development of safe lifting behavior specifically in those working categories that are exposed to a high risk of injury. Therefore, the central issue is not “make workers practicing lifting” but instead “create the condition for promoting deliberate practice in the workplace with the goal of improving one's own lifting behavior.” We contend next that augmented feedback (i.e., feedback from an external source) holds the key for achieving such goal, as it will create suitable training conditions whilst allowing a worker to perform work duties as they normally would.

### Augmented Feedback to Promote Deliberate Practice for Training Lifting in the Workplace—Design and Research Issues

Creating suitable training conditions to apply deliberate practice in the workplace is quite a tricky affair, as workers have a tight schedule to follow and do not have spare time to dedicate to additional practice. According to Ericsson (2021), a training condition should include these main features to promote deliberate practice: “(i) the task must be well defined with a clear goal and be fully understood by participant, (ii) the participants need to be able to perform the task by themselves, (iii) the participants need to gain immediate informative and actionable feedback on each performance of the practice task that allow them to make appropriate adjustments to improve, (iv) the participants need to be able to repeatedly perform the same or similar tasks, and (v) the practice task must be designed and performed in accordance with individualized instructions and guidance of a teacher.” Items i, ii, and iv are inherent to lifting in the workplace, and the implementation of augmented feedback will contribute to achieve items iii and v. For example, research in medical training has shown the benefits of properly integrating feedback within a deliberate practice framework to promote the development of surgery skills (e.g., Blackhall et al., 2019; Higgins et al., 2021).

Augmented feedback (we will refer to it as feedback from now on) is considered a key component for motor skill learning (Swinnen, 1996; Sigrist et al., 2013a); some researchers even consider it a condition *sine qua non* for learning (Bilodeau, 1966). Feedback can augment information naturally available to the senses (i.e., intrinsic feedback from vision, acoustics and haptics) and/or provide additional information related to the task at hand (e.g., accuracy of a movement). It is instrumental in directing a learner's attention to critical movement- and goal-related information (e.g., proprioceptive information), facilitating a learner's exploration and stabilization of goal-relevant movement solutions (Newell, 1991). The design of feedback (strategies) for promoting deliberate practice is a complex task, since the benefits of feedback depend on a complex interplay of situational and individual factors (e.g., Narciss, 2008, 2012). The main challenge is to design an effective, yet feasible feedback strategy that is accepted by workers and can be implemented in the workplace. Importantly, feedback has to fulfill the requirements for promoting deliberate practice, i.e., feedback has to be *informative, actionable, and individualized*.

Mere practice of the lifting movement is not a satisfactory condition, and feedback can remind a worker to pay attention to how they are lifting and, if necessary, encourage them to focus on ways to improve the movement. For example, alarm-based auditory feedback (e.g., a beep) triggered when spine bending is excessive will tell a worker that something is not correct and will direct their attention to how they are controlling the spine during lifting. *Feedback has to be informative and actionable*. Feedback can provide information on the quality of the lifting movement and on how certain parts of the movement can be changed to improve the movement. For example, auditory feedback that increase in frequency according to the bending of the spine will provide information about the degree of bending, and consequently on the amount of required change in movement. Furthermore, according to a pre-determined range of suitable movement parameters, haptic feedback can indicate a worker to increase flexion of the knees to in turn decrease flexion of the lumbar area. *Feedback has to be individualized* and tailored to each single worker, to encourage changes in movement that are functional to the worker's characteristics and the environment they operate in. For instance, feedback on spine bending should be presented as a percentage of the worker's maximal flexion ability, or hints for movement change should be specific to the worker's intrinsic modality of movement (e.g., should not “tell” a worker with low hamstring flexibility to increase flexion of the hips).

Ultimately, feedback has great potential to facilitate the implementation of an embodied and embedded training intervention directly in the workplace. It can promote workers developing their own knowledge and performance of the lifting movement(s) suitable for the characteristics of the context they work in. With appropriately-designed feedback strategies, workers will be able to accomplish their working duties as they normally would (e.g., move boxes in a warehouse or lift luggage at the airport), while concurrently direct part of their effort and attention to improving bit-by-bit their lifting movement. The motor control and learning literature can inform the design and implementation of such strategy (Bilodeau, 1966; Magill and Anderson, 2012). Research conducted in the laboratory can provide critical information on the benefits and boundary conditions of different feedback strategies. Feedback content and modality are critical aspects of a feedback strategy.

#### Feedback Content

An analysis of the information that guides action in a certain task and the movement patterns that correlate with successful performance can inform the design of feedback content. A common strategy is to observe expert performance and examine what features differentiate experts and novices (i.e., the expert performance approach; Ericsson, 2003), and consequently infer what key task-related information characterizes a functional action (Oppici et al., 2021). Feedback content can be designed accordingly.

In the context of maintaining a safe neutral spine during lifting, an enhanced proprioception of lumbar spine position is key (i.e., position sense; Proske and Gandevia, 2012). Individuals with low back pain have a lower ability to perceive the position of their lumbar spine—proprioception deficit—than healthy ones (Willigenburg et al., 2013; Tong et al., 2016). An accurate perception of proprioceptive information regarding one's own spine posture can guide coordination of the different body parts to achieve the goal of reducing lumbar spine flexion. However, given that proprioception accuracy decreases when the trunk is flexed, which typically happens during lifting a box from the ground (Wilson and Granata, 2003; Gade and Wilson, 2007), accurate proprioception of the spine is particularly challenging during lifting movements. Feedback can be instrumental here. It can provide information on spine position, and in turn promote workers tuning their perception to proprioceptive information regarding one's own spine posture. This will be particularly relevant for critical flexed positions during lifting. An increase in proprioception is then expected to support a reduction in spine flexion.

Previous research has shown that augmenting lumbar spine parameters (e.g., lumbar flexion angle) during lifting is effective in reducing peak lumbar flexion (Agruss et al., 2004; Kernozek et al., 2006; Lavender et al., 2007; Matheve et al., 2018; Pinto et al., 2018; Boocock et al., 2019; Lorenzoni et al., 2019; Punt et al., 2020). Reduction in peak spine flexion observed during practice was maintained when feedback was removed (i.e., transfer test) in Agruss et al. (2004), Kernozek et al. (2006), Lavender et al. (2007), Matheve et al. (2018), Pinto et al. (2018), and Punt et al. (2020), indicating that learning was starting to occur, but none of the studies included a long-term retention test and it is therefore unclear whether this performance improvement can persist over time. Nevertheless, these studies indicate that providing augmented feedback on how much an individual flexes their spine is effective in reducing spine flexion.

The reduction of lumbar spine flexion in the studies above was obtained by redistributing flexion to the lower body. Concurrent to a reduction in peak lumbar flexion angle, peak flexion angle increased at the hip and at the knee joints (Matheve et al., 2018; Pinto et al., 2018; Boocock et al., 2019; Punt et al., 2020). In other words, participants bent their knee more and tilted their hip forward when provided with the augmented feedback. This redistribution of spine flexion along the lower body, known as spine sparing technique (Makhoul et al., 2017), allows people to flex their trunk and lower their center of mass to grab a box from the ground while reducing flexion of the lumbar spine. This feedback strategy does not prescribe individuals how to specifically change movement coordination to reduce spine flexion, but rather provides information on how much they are bending the spine. Thus, individuals are free to modify their coordination to achieve the goal of reducing spine flexion. This aligns well with the idea (expressed in previous section) that a training intervention should create the condition for workers to develop their own understanding of and solution(s) to improve movement to reduce their spine flexion. Furthermore, this strategy likely results in the exploration and stabilization of different movement solutions that can adapt to changes in working circumstances (for examples see Pacheco et al., 2019; Garofolini et al., 2020).

#### Feedback Modality

Augmented feedback on lumbar spine flexion can be delivered using different modalities, and an interesting open issue is how different modalities influence a reduction in spine flexion. Feedback on lumbar spine flexion has been delivered using visual (Matheve et al., 2018), auditory (Kernozek et al., 2006; Lavender et al., 2007; Boocock et al., 2019; Lorenzoni et al., 2019; Punt et al., 2020), verbal (Agruss et al., 2004) and tactile (Pinto et al., 2018) modalities. The visual modality has come under scrutiny (Sigrist et al., 2013a) and it may not represent the best solution in tasks that require individuals to use vision for guiding goal-directed movement, such as lifting and placing a box onto a shelf. In this sense, augmented visual feedback may overload the visual system and distract workers. Similarly, the verbal modality does not seem feasible in an occupational context, as it would constantly require the presence of a trainer. The auditory and tactile modalities represent interesting avenues and can be applied in an occupational context. Both modalities provide augmented information using a channel different to vision and they present important advantages with respect to motor learning.

##### Auditory Feedback

The auditory feedback modality has been shown to effectively promote a reduction of lumbar spine angle during lifting (Kernozek et al., 2006; Lavender et al., 2007; Boocock et al., 2019; Lorenzoni et al., 2019; Punt et al., 2020). In this feedback modality, sound is used to augment information about movement, e.g., spine flexion angle. Information is typically delivered through alarm-based sound, whereby a sound is triggered when a movement parameter exceeds a pre-set threshold (e.g., 20% of spine bending), and movement sonification, which maps sound onto movement and sound changes according to movement (e.g., sound frequency increases according to increase in spine flexion). While sonification of a movement parameter or movement error seems to be more beneficial than an alarm-based sound for augmenting postural parameters (Dozza et al., 2011; Ghai and Ghai, 2018; Ghai et al., 2019), previous research has primarily examined the effect of alarm-based sound on reducing lumbar spine flexion. There is therefore room for further investigating how movement sonification influences lumbar spine flexion reduction. In addition to using a channel different to vision, an important advantage of the auditory feedback modality is that it limits the risk of incurring into the guidance effect (Dyer et al., 2017; Hasegawa et al., 2017; Ghai et al., 2019). The guidance effect occurs when augmented feedback substitutes task-relevant information, and improvement in movement disappears when feedback is removed (Salmoni et al., 1984). Auditory feedback on the contrary promotes a learner tuning their perception to the augmented information, e.g., lumbar spine angle, and improvement should persist when feedback is removed. On the other hand, however, the auditory modality requires a learner to map sound onto their movement, which may be problematic in individuals with a low perception of their body/movement and thus it may increase the learning period (Sigrist et al., 2013b).

##### Tactile Feedback

Tactile represents another suitable modality for augmenting spine flexion during lifting, and it can be particularly relevant for the occupational sector. It may represent an easy-to-implement, cheap and yet effective strategy. Tactile refers to sensory information perceived by mechanoreceptors situated under the skin, which are part of the proprioceptive system (Proske and Gandevia, 2012). Tactile feedback is applied directly on the skin and can be delivered through skin deformation, vibration or force feedback. A sport leukotape (firm tape) placed on the lumbar extensor muscles has been shown to improve spine proprioception and reduce lumbar spine flexion via skin stretching (Pinto et al., 2018). In short, the tape stretches the skin when the lumbar area flexes, and this stretch augments information about spine flexion (Beaudette et al., 2018). The higher the skin stretch the higher the flexion of the spine. Importantly, skin sensitivity to stretching improves in flexed position, so information from the tape can be better perceived in the critical part of the lifting movement (Beaudette et al., 2017). A critical advantage of using tape for providing tactile feedback is that information is provided directly on the area of interest and a learner does not need to map augmented information onto movement. On the other hand, a tape may “substitute” proprioceptive information and may guide movement. Learners would perceive proprioceptive information only with the tape on and performance may decrease when it is removed.

#### Role of Individual Differences

An often overlooked design and research issue of the effect of augmented feedback on learning is how different individuals respond to feedback. Seminal research in motor learning has shown how a learner's initial movement coordination patterns influenced their learning (Zanone and Kelso, 1992, 1997; Kostrubiec et al., 2012). Some research has also investigated how a learner's skill level interacts with feedback effect using relatively simple tasks (Magill, 1994). To the same extent, different initial movement capabilities may influence how individuals use different feedback modalities to regulate their movement. Some individuals may benefit more from a feedback modality, while others may prefer another modality. This would mean that training programs need to be flexible and suit the characteristics of the different learners. As often happens in motor learning, one size does not fit all, and training has to be tailored to the learner's current abilities. This issue however has rarely been considered in the context of teaching the lifting movement in the occupational context.

#### The Distinction of Feedback Effects in Terms of Guiding Performance and Promoting Learning

An important research issue to consider is the distinction between learning and performance effect, which has implications for the design and assessment of training interventions. Performance is observable, while learning is not (it is abstract); therefore, learning has to be inferred from performance (Wulf et al., 2010; Magill and Anderson, 2017). For inferring that learning has occurred, performance has to improve, persist, adapt, and transfer. For example, we can claim that a certain augmented feedback promotes learning only if performance improvement persists when the feedback is removed (Salmoni et al., 1984). The *guidance effect* (i.e., performance decreases when feedback is removed) occurs when augmented feedback “substitutes” task-relevant information, and learners regulate their action and become dependent on the augmented information rather than on task-relevant information (Dyer et al., 2017). As such, the design of a training intervention should include a retention or transfer test to properly assess learning, and should focus on educating a learner's attention to the key information (e.g., spine proprioception) that guides movement and eventually learning. Furthermore, the learning-performance distinction sets apart learning device/strategy and assisting device/strategy. For instance, lumbar supporting devices, such as belts and exoskeletons have been shown to reduce trunk and lumbar flexion; however, their effect on learning has been hardly if ever examined in a retention/transfer test, and they can be considered as a learning device only if proper instructional strategies are put in place to direct a learner's attention to the information that the belt provides on the skin.

## Benefits and Constraints of Feedback Modality on Reducing Lumbar Spine Flexion in a Repetitive Lifting Task: A Pilot Study

In summary, both the auditory and feedback modalities have been shown to effectively reduce lumbar spine flexion during lifting. The two different feedback modalities have different advantages and disadvantages with regards to promoting motor learning. Previous research has mainly compared tactile and auditory feedback using relatively simple tasks (Sigrist et al., 2013a); for example, Scott and Gray (2008) has shown a slightly lower reaction time with tactile than auditory feedback. It is however unclear how these two feedback modalities influence learning of a complex task. In training lifting, studies have focused on one feedback modality only, and no study has compared the effectiveness of the two feedback modalities. Furthermore, it is unclear whether and how a learner's initial movement capabilities can influence the effect of these feedback modalities.

We conducted a pilot study comparing the effect of tactile and auditory feedback modalities on reducing spine flexion during a repetitive lifting task. The aim of this study was to gather initial insights into how tactile and auditory feedback influence spine control during lifting in the laboratory, which can then inform a larger applied learning study. In this within-subject experiment, participants lifted a 7.5-kg box under three conditions: (i) tactile feedback, a sport Leukotape was applied to the right and left lumbar extensor muscles, from T12 to the sacrum (similar to Pinto et al., 2018), (ii) auditory feedback, a continuous real-time sound was mapped onto lumbar spine flexion (similar to Lorenzoni et al., 2019), and (iii) control, no feedback was provided. In each condition, participants performed 30 lifting practice trials and after 5 min five retention/transfer trials (i.e., without feedback). The control condition was always performed first, and the two feedback conditions were randomized and counter-balanced between participants. We hypothesized (i) both feedback conditions to reduce peak lumbar spine flexion relative to the control condition in the practice trials, (ii) both feedback conditions to maintain a superior performance in retention trials relative to control condition (i.e., learning effect), (iii) a higher reduction of performance from acquisition to retention trials in the tactile relative to the auditory condition (i.e., guidance effect), and (iv) tactile feedback to be more effective than auditory feedback in participants with high spine flexion during the control condition (i.e., bad initial spine posture). Furthermore, we explored how the two feedback conditions influenced changes in participants' coordination for reducing lumbar flexion angle.

### Methods

#### Participants

Twenty adults (30 ± 6 years old, 1.78 ± 0.1 m, 75 ± 18 kg, 35% females) were recruited from a University student population. Individuals were excluded from the study if they had back injury or pain in the last year, undergone spinal surgery, had any cardiovascular, neurological or musculoskeletal condition at the time of the study, were allergic to adhesives. This sample size was based on a previous study with similar within-factors design (Pinto et al., 2018). All participants gave informed consent and the research team's University Ethics Committee approved the study.

#### Experimental Task, Design, and Procedure

**Figure 1:**
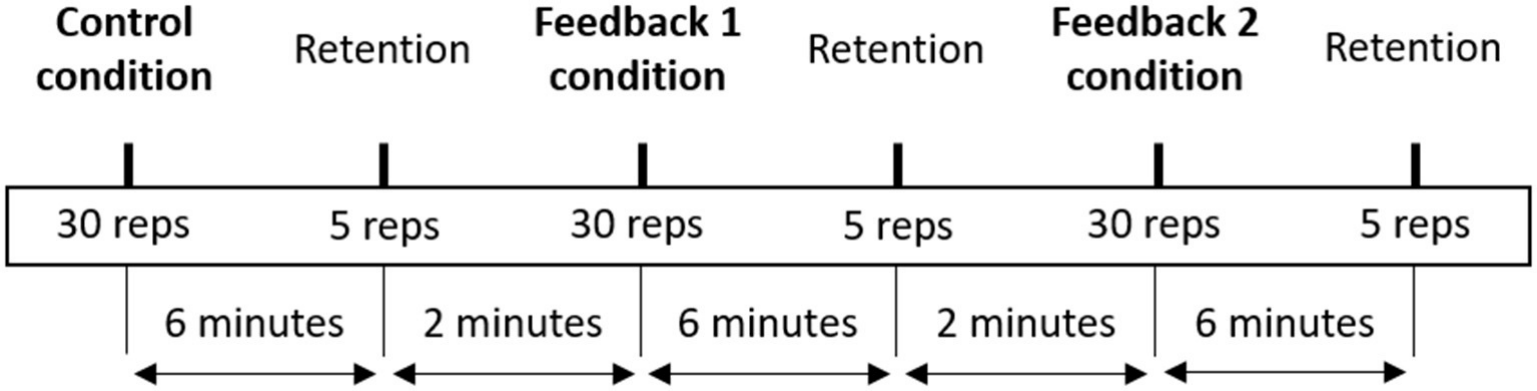
Schematic overview of the experimental procedure.

The experimental task consisted of lifting a 7.5-kg box from ground to knuckle height, keeping the arms extended and relaxed, and then lowering the box back to the ground. The movement started with a standing position and was comprised of two phases: (1) bend down, grab the box handle, and lift the box; (2) lower the box, position it on the ground, and stand back up to starting position without the box. The box had two handles positioned 25 cm above the ground, and participants were instructed to align their toes with the closest box side. To ensure consistency within and between participants, lines on the floor marked the position of box and participant's toes. Three lifting conditions were designed: two feedback conditions (tactile and auditory) and a control condition (no feedback). The control condition was always performed first, and the order of the two feedback conditions was randomized using a number generator and counter-balanced between participants. In each condition, participants first performed 30 repetitions (acquisition phase) at 10 reps per minute, and after 6 min 5 retention repetitions (retention phase). Feedback was present during the acquisition phase and removed during the retention phase. The conditions were interspersed with a 2-min break (Figure 1). A visual metronome, with a 20 beats/min tempo, provided the lifting rhythm, and each visual cue (i.e., LIFT and LOWER) indicated the start of each movement phase, which means that 10 lifting-lowering repetitions were performed in 1 min. The visual cues were displayed on a screen positioned on the floor, 3 meter in front of participants, to avoid constraining their head/spine movement. This lifting tempo was designed to standardize the lifting rhythm across participants and conditions.

Upon their arrival at the laboratory, participants were provided with an explanation of the experimental procedure (without specific hints on hypotheses) and were fitted with retro-reflective markers. Then, they performed a 5-min customized warm up which included spine mobilization (guided by an experimenter) and squatting movements at their own tempo using their preferred technique. After the warm up, participants received instruction on the lifting movement: “the task requires you to lift the box from ground to knuckle height and then place it back to the ground. During the movement, maintain your feet in the marked position, keep your arms extended and relaxed, and avoid bending your back.” To ensure consistency, a designated experimenter provided instructions to all participants, following a written script. No demonstration was provided to avoid influencing participants' lifting movement. Participants were then allowed to perform 5 practice trials. In the control condition, participants did not receive any further instruction or feedback. In the feedback conditions, they received the assigned feedback and further instruction on the feedback procedure.

#### Biomechanical Model and Kinematic Measures

A ten-camera motion capture system (Qualisys AB, Gothenburg, Sweden) recorded 3-dimensional kinematics, sampling at 100 Hz. Forty-six reflective markers were applied to participant's skin to track the position and orientation of the trunk (10 markers), pelvis (6 markers), thighs (6 markers each), shanks (6 markers each), and feet (3 markers each). After a static trial, 10 calibration markers were removed. Three markers were attached to the box. Importantly, the markers on the spine were attached in correspondence to C7, T7, T12, L2, L4, and sacrum. T12, L2, L4, and sacrum were used to track lumbar spine flexion for the auditory feedback.

**Figure 2:**
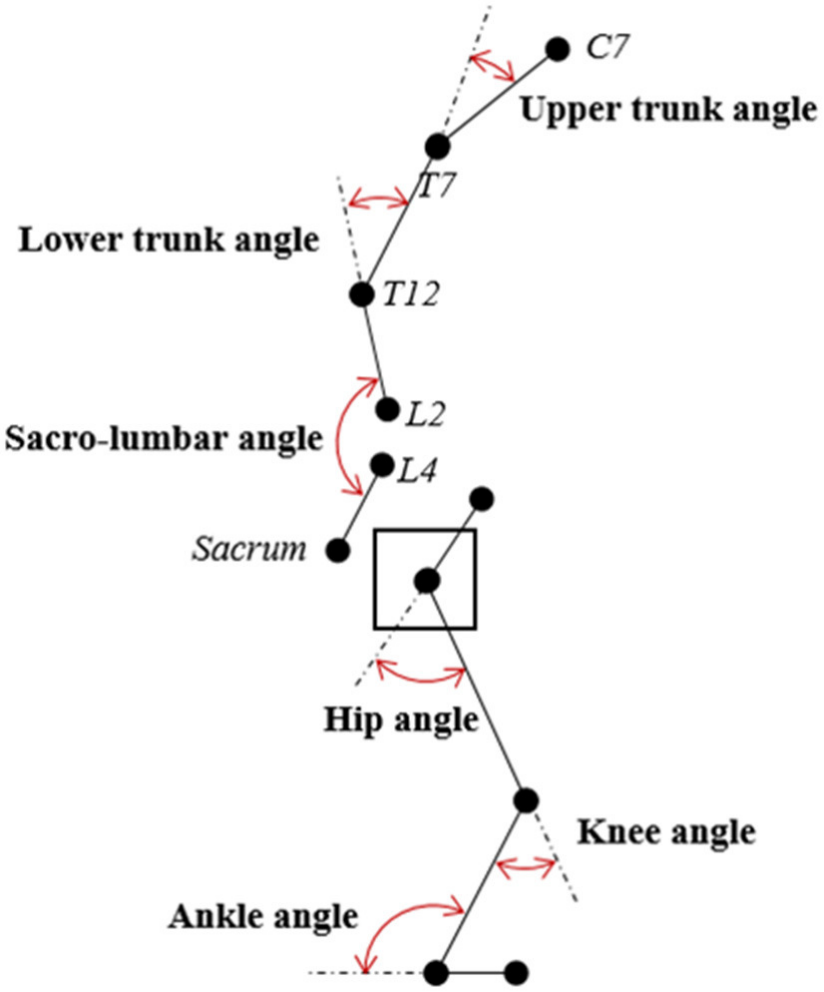
Schematic representation of the body angles.

Data was exported for analysis to Visual3D software (C-Motion, Inc.). Markers trajectories were low-pass filtered (15 Hz) before computing the angles of interest. The sacro-lumbar angle was the main outcome of interest and was computed as planar (YZ) angle between the T12–L2 and the L4-sacrum segments using the L4-sacrum segment as reference (Figure 2). Furthermore, upper trunk and lower trunk angles were computed as planar (YZ) angles between the C7–T7 and the T7–T12 segments, and between the T7–T12 and the T12–L2 segments respectively. The three-dimensional motions of knee, ankle, and hip were investigated through positioning of the segments with respect to each other. Joint rotation was calculated around the x axis on the sagittal plane (flexion/extension) using the Cardan sequence X–Y–Z (C-Motion, 2020). All angles were normalized by the angles recorded during the static pose. The start and the end of the lift were defined by identifying the lowest (start) and highest (end) values of a box marker's vertical position. Peak angles were then extracted as the maximum value between the beginning and the end of the lift.

#### Tactile Feedback

Sport leukotape was applied to participant's skin overlaying the left and right lumbar extensor muscles, from the 12th thoracic vertebra to the sacrum, from a standing position with neutral spine. Participants were given the instruction “You will feel the tape stretching if you bend your back. Avoid bending your back and avoid stretching the tape,” consistent with previously published procedure (Pinto et al., 2018). Participants had the chance to make some movement with their back and feel the stretching of the tape before starting the lifting repetitions. Leukotape was removed at the end of this condition.

#### Auditory Feedback

Lumbar spine flexion feedback was presented in real-time via the auditory modality during the lifting movement. The position of spine markers, provided by the motion capture systems' API, was accessed from MATLAB. The Euclidian distance between T12, L2, L4, and Sacrum was calculated in MATLAB and was used as a proxy of spine flexion, following published procedure (Lorenzoni et al., 2019). The sum of the distances represented the instantaneous length of the lumbar spine (SB). Then, the instantaneous spine flexion was calculated as a percentage value as follow: Flexion = (SB—neutral SB)/(maximally flexed SB—neutral SB). Neutral SB was calculated by asking participants to stand still, fixating a spot in front of them, while maximally flexed SB was calculated by asking participants to maximally flex their spine (the experimenter helped them finding this position). The underlying assumption of this procedure is that an increase in spine length reflects a flexion in the lumbar area.

The proxy of spine flexion data was sent from MATLAB to Pure Data via Open Sound Control protocol. A parametric sound generator was implemented in Pure Data. The sound characteristics were chosen to enable participants to intuitively distinguish a bent from a straight spine flexion. Applying amplitude modulation and increasing the modulation frequency with increasing spine flexion was selected for this purpose. The fluctuation sensation has the advantage of allowing the listener to assess the modulation frequency easily and thus offers a very salient cue on the degree of “unevenness.” Fluctuation strength is a well-defined sound property (Zwicker and Fastl, 2013). The perceived fluctuation strength increases from 0% for an unmodulated 1 kHz tone to 100% for a modulation frequency of 8 Hz and decreases again above that frequency. Thus, in order to maximize the perceived contrast, neutral SB was encoded as an unmodulated 440 Hz tone, while maximally flexed SB was encoded as 8 Hz modulation frequency. The implemented sound generator produces a sound accordingly throughout the whole lifting movement. The modulation frequency changed dynamically according to the spine flexion proxy in real time.

Participants were instructed “You will hear a continuous sound. The sound increases in modulation frequency if you bend your back. Avoid bending your back and keep the sound similar to the neutral standing position.” Participants had the chance to make some movement with their back and hear how the sound was mapped on their spine flexion. This procedure was comparable to the tactile condition.

#### Statistical Analysis

Linear mixed modeling (LMM) was computed on all the dependent variables with a normal distribution of their residuals, except the peak sacro-lumbar angle, which had a non-normal distribution of residuals. For peak sacro-lumbar angle, generalized linear mixed modelling (GLMM) with log transformation of the target variable and robust covariance was adopted. GLMM largely improved the fit of the model (e.g.,−2 Log-Likelihood: 192 with GLMM vs. 2905 with LMM) and the residuals were normally distributed (skewness [0,08] and kurtosis [0,12]; Kolmogorov-Smirnov and Shapiro-Wilk tests were not significant). Residuals were checked after each analysis and they were normally distributed in all analyses.

GLMM was computed on peak sacro-lumbar angle, and LMM was computed separately on the following dependent variables: upper trunk, lower trunk, hip, knee, and ankle flexion angle. Participants were always considered as random factors (intercept was not included because covariance was 0 and it caused the model not to converge), while fixed and repeated factors varied depending on the analysis of interest. For the acquisition phase, the model was computed on the acquisition trials with condition (control, audio, tactile) and trial block (1–5, 6–10, etc.) as fixed and repeated factors. To assess learning effect, the model was computed on the retention trials with condition (control, audio, tactile) as fixed and repeated factor. To assess the guidance effect, the model was computed on acquisition and retention trials with trial block (1–5, 6–10, …, retention) as fixed and repeated factor in each condition individually.

To assess the influence of initial spine posture (i.e., spine flexion during the control condition) on feedback effect, the model was computed for the sacro-lumbar angle on the acquisition trials with baseline (control condition) and condition (audio, tactile) as covariates, repeated measures on the condition factor. Furthermore, to test how a more or less flexed initial posture in the baseline influenced participants' performance in the two feedback conditions, one standard deviation (of the baseline performance) was added (decrement in performance) or subtracted (improvement in performance) to participants' baseline, and a linear mixed model similar to previous was computed.

*Post hoc* pairwise comparisons were computed using Bonferroni adjustment for multiple comparisons. Statistical significance was set at *p* < 0.05. Cohen's d_z_ was calculated on the *Post hoc* pairwise comparisons to estimate the magnitude of effect. Cohen's d_z_ takes into account dependency between the repeated measures by including the between-measures correlation into the computation (Lakens, 2013). The effect magnitude was classified trivial (*d* < 0.2), small (0.2 < *d* < 0.5), moderate (0.5 < *d* < 0.8) and large (*d* > 0.8). Data analysis was performed using SPSS statistical software (version 27.0. Armonk, NY: IBM Corp.).

### Results

All 20 participants completed data collection and were included in the analysis. A preliminary analysis showed that the effects were the same for the lifting and lowering phases, so the effects are reported for the lifting phase only.

Descriptive statistics of the main outcome—peak sacro-lumbar flexion angle—across conditions and trial blocks is presented in Table 1.

**Table 1:** Peak sacro-lumbar flexion angle expressed as median and interquartile range (Q1–Q3) across the experimental conditions and trial blocks.

		**Control**		**Auditory**		**Tactile**	
	**Trial block**	**Median**	**IQR**	**Median**	**IQR**	**Median**	**IQR**
Acquisition phase	1–5	20.3	12–31	16.1	8–21	14.4	9–22
	6–10	18.8	11–30	13.9	8–25	14.0	10–24
	11–15	21.5	11–30	15.7	9–23	14.4	9–24
	16–20	22.0	11–31	15.8	9–23	14.8	12–24
	21–25	22.1	12–31	16.0	11–25	16.4	12–25
	26–30	21.1	12–30	16.6	9–26	15.4	13–26
	Overall	21.5	11–31	15.7	9–25	15.0	11–25
Retention	1–5	21.5	11–31	17.8	10–23	18.7	10–26

*All 20 participants were included in the analysis*.

#### Acquisition Phase

##### Sacro-lumbar Peak Flexion Angle

Results showed a statistically significant effect of condition (*F*_[2,57]_ = 19.68, *p* < 0.01), no significant effect of trial (*p* = 0.40) and condition*trial (*p* = 0.46). *Post hoc* analysis showed that flexion angle was significantly higher in the control than the auditory (*p* < 0.01) and tactile (*p* < 0.01) conditions; the angle was higher in the auditory than tactile condition (*p* < 0.01) (Table 2; **Figure 4A**).

**Table 2:** Pairwise comparisons in the acquisition phase.

	**Sacro-lumbar**			**Upper trunk**			**Lower trunk**		
	***p* value**	**Δ; LL–UL**	**Cohen's d_**z**_**	***p* value**	**Δ; LL–UL**	**Cohen's d_**z**_**	**p value**	**Δ; LL–UL**	**Cohen's d_**z**_**
Control vs. auditory	<0.01	2.3; 1.2–3.4	0.3	<0.01	0.8; 0.3–1.2	0.5	<0.01	1.8; 0.8–2.8	0.5
Control vs. tactile	<0.01	5.6; 2.9–8.3	0.4	0.12	0.3; −0.1–0.6	0.2	<0.01	2.8; 1.9–3.6	0.8
Auditory vs. tactile	<0.01	3.4; 1.6–5.1	0.3	<0.01	−0.5; −0.8–−0.2	0.5	<0.01	0.9; 0.3–1.6	0.3
	**Hip**			**Knee**			**Ankle**		
	***p*** **value**	Δ**; LL–UL**	**Cohen's d**_**z**_	***p*** **value**	Δ**; LL–UL**	**Cohen's d**_**z**_	***p*** **value**	Δ**; LL–UL**	**Cohen's d**_**z**_
Control vs. auditory	0.35	0.9; −0.5–2.3	0.1	<0.01	8.2; 1.9–14.4	0.5	1.0	0.4; −1.2–2.1	0.1
Control vs. tactile	<0.01	−4.9; −3.1–−6.6	0.6	<0.01	11.6; 6.5–16.7	0.5	0.5	0.9; −0.7–2.47	0.1
Auditory vs. tactile	<0.01	−5.8; −7.9–−3.6	0.8	<0.01	3.4; 0.4–7.1	0.2	1.0	0.4; −1.0–1.9	0.1

*p value, mean difference (Δ) with 95% Lower and Upper confidence limits (LL and UL), and Cohen's d_z_ are presented. All 20 participants were included in the analysis*.

##### Secondary Outcomes

**Figure 3:**
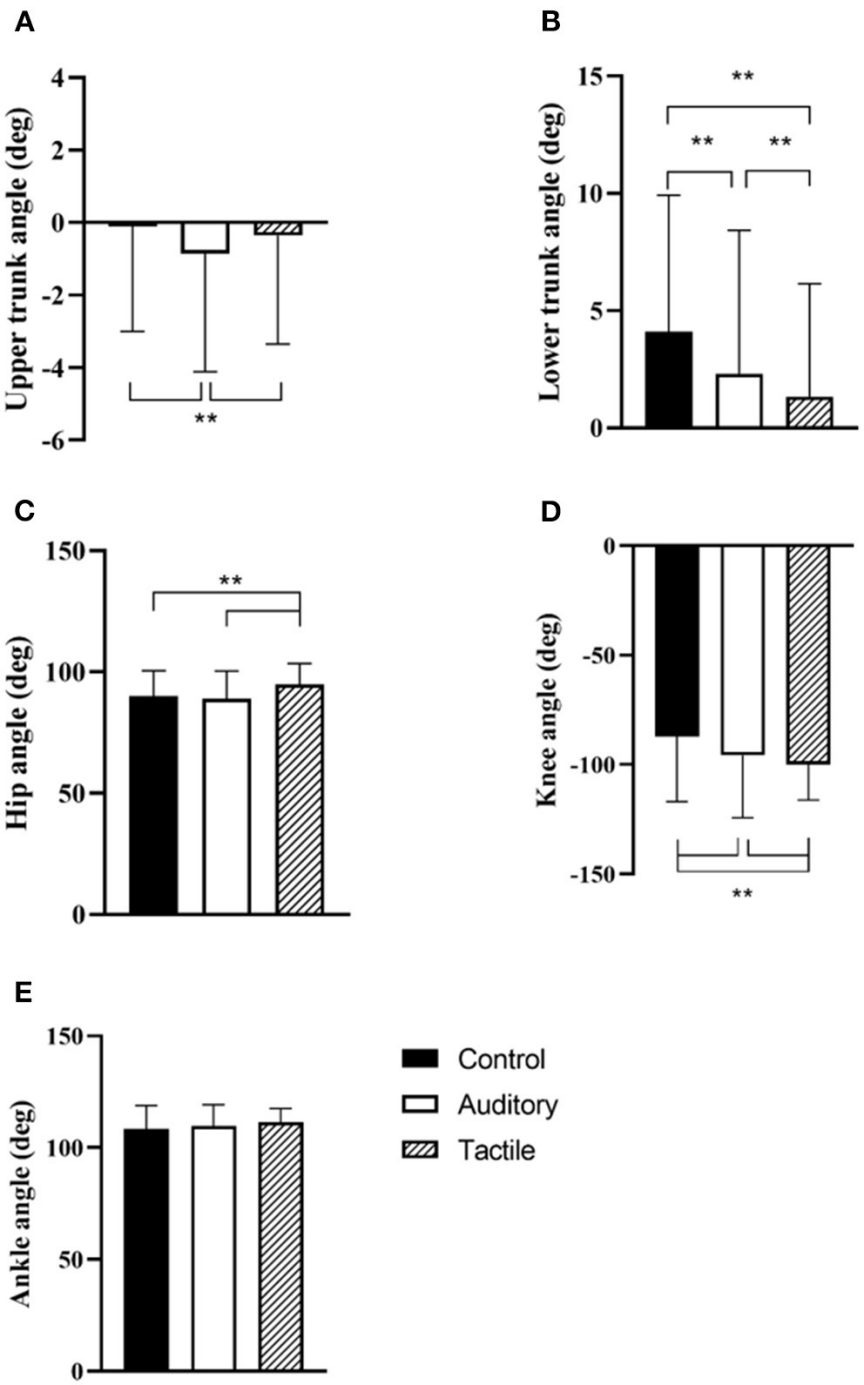
Condition effect in the acquisition phase for peak upper trunk **(A)**, lower trunk **(B)**, hip **(C)**, knee **(D)**, and ankle **(E)** flexion angles. ** indicates *p* < 0.01.

There was a statistically significant effect of condition for peak upper trunk (*F*_[2,342]_ = 12.44, *p* < 0.01), lower trunk (*F*_[2,342]_ = 38.66, *p* < 0.01), hip (*F*_[2,342]_ = 24.69, *p* < 0.01), and knee (*F*_[2,342]_ = 15.18, *p* < 0.01) flexion angles. No significant effect of trial nor condition*trial in these angles. *Post hoc* analysis showed that upper trunk angle was significantly lower in the auditory than the control (*p* < 0.01) and tactile (*p* < 0.01) conditions; lower trunk angle was significantly lower in the tactile than the control (*p* < 0.01) and auditory (*p* < 0.01) conditions, and in the auditory than the control (*p* < 0.01) condition; hip angle was significantly higher in the tactile than the control (*p* < 0.01) and auditory (*p* < 0.01) conditions; knee angle was significantly lower in the control than the auditory and tactile (*p* < 0.01) conditions, and in the auditory than the tactile (*p* < 0.01) condition (Table 2; Figure 3).

In peak ankle flexion angle, there was only a statistically significant effect of trial (*F*_[5, 79]_ = 3.02, *p* = 0.02). The intercept of random effect was 77 ± 25. *Post hoc* analysis showed that peak ankle flexion angle approached significance in trial 3 relative to trial 1 (*p* = 0.09) (Table 2; Figure 3).

#### Learning Effect

##### Sacro-lumbar Peak Flexion Angle

**Figure 4:**
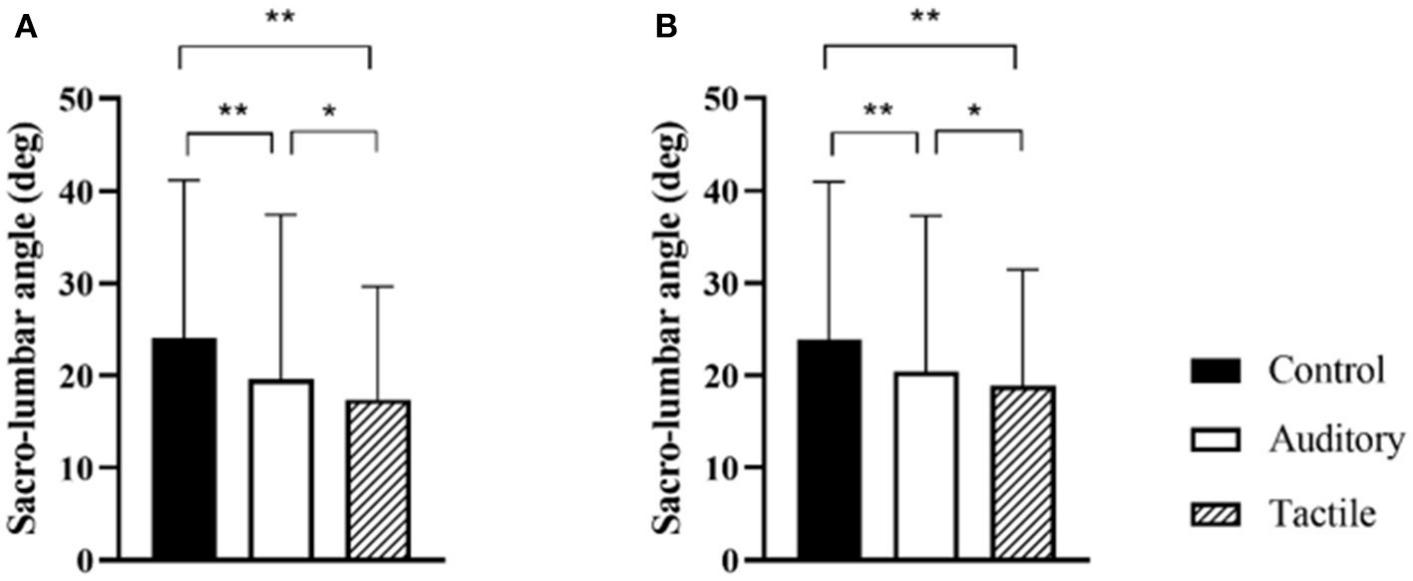
Condition effect for peak sacro-lumbar angle in the acquisition and retention phases (**A,B** respectively). * and ** indicate *p* < 0.05, and *p* < 0.001 respectively.

Results showed a statistically significant effect of condition (*F*_[2,57]_ = 13.28, *p* < 0.01). *Post hoc* analysis showed that spine angle was significantly higher in the control than the tactile (*p* < 0.01) and auditory (*p* = 0.048) conditions; the angle was trivially higher in the auditory than the tactile condition (*p* = 0.048) (Table 3; Figure 4B).

**Table 3:** Pairwise comparisons in the retention phase (learning effect).

	**Sacro-lumbar**			**Upper trunk**			**Lower trunk**		
	***p* value**	**Δ; LL–UL**	**Cohen's d_**z**_**	***p* value**	**Δ; LL–UL**	**Cohen's d_**z**_**	***p* value**	**Δ; LL–UL**	**Cohen's d_**z**_**
Control vs. auditory	0.048	2.1; 0.2–4.2	0.4	1.0	0.6; −1.0–2.3	0.2	0.07	2.1; −0.1–4.3	0.6
Control vs. tactile	<0.01	4.6; 1.8–7.5	0.6	1.0	0.5; −1.0–2.1	0.2	0.03	2.2; 0.2–4.2	0.6
Auditory vs. tactile	0.048	2.5; 0.02–5.0	0.3	1.0	−0.1; −0.8–0.6	0.1	1.0	0.1; −1.5–1.6	0.1
	**Hip**			**Knee**			**Ankle**		
	**p value**	**Δ; LL–UL**	**Cohen's d**_**z**_	**p value**	**Δ; LL–UL**	**Cohen's d**_**z**_	**p value**	Δ; **LL–UL**	**Cohen's d**_**z**_
Control vs. auditory	1.0	−1.0; −4.8–2.8	0.1	0.4	10.1; −7.3–27.6	0.6	0.7	−2.1; −6.8–2.6	0.3
Control vs. tactile	0.12	−4.2; −9.0–0.7	0.5	0.07	12.7; −0.8–26.1	0.5	0.07	−4.2; −8.6–0.2	0.5
Auditory vs. tactile	0.3	−3.1; −7.7–1.5	0.4	1.0	2.6; −10.7–15.8	0.1	0.5	−2.1; −5.9–1.6	0.3

*p value, mean difference (Δ) with 95% Lower and Upper confidence limits (LL and UL), and Cohen's d_z_ are presented. All 20 participants were included in the analysis*.

##### Secondary Outcomes

There was a statistically significant effect of condition for peak lower trunk flexion angle only (*F*_[2,21]_ = 4.26, *p* = 0.03). *Post hoc* analysis showed that the angle was significantly higher in the control than the tactile (*p* = 0.03) condition, and a trend of being higher than the auditory (*p* = 0.07) condition (Table 3).

#### Guidance Effect

##### Sacro-lumbar Peak Flexion Angle

**Figure 5:**
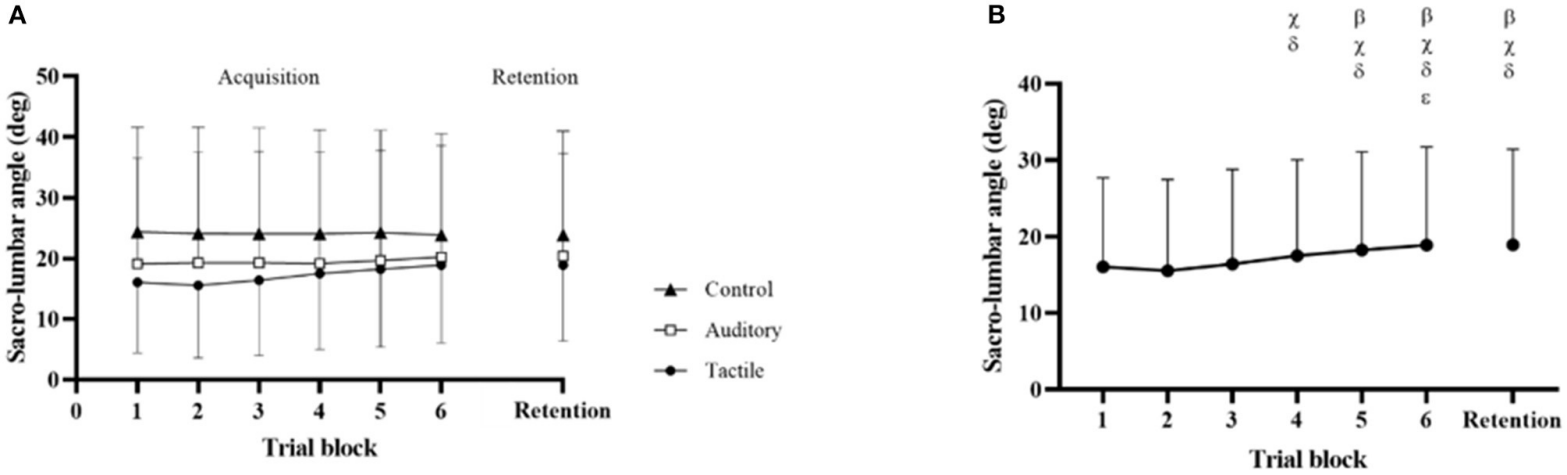
Condition × Trial effect across the 3 conditions **(A)**, and trial effect in the tactile condition **(B)**. β, χ, δ, and ε indicate significant difference with block 1, block 2, block 3, and block 4 respectively.

Results showed a statistically significant trial effect in the tactile condition (*F*_[6,133]_ = 16.62, *p* < 0.01), but no trial effect in the control (*p* = 0.8) and the auditory (*p* = 0.3) conditions. *Post hoc* analysis showed that spine angle was significantly higher in retention block than block 1 (*p* = 0.03), block 2 (*p* < 0.01), and block 3 (*p* = 0.02); block 6 was higher than block 1 (*p* < 0.01), block 2 (*p* < 0.01), block 3 (*p* < 0.01), and block 4 (*p* < 0.01); block 5 was higher than block 1 (*p* = 0.03), block 2 (*p* < 0.01), and block 3 (*p* < 0.01); block 4 was higher than block 2 (*p* < 0.01) and block 3 (*p* < 0.01) (Figure 5).

##### Secondary Outcomes

There was a significant trial effect in the tactile condition for the peak lower trunk flexion angle (*F*_[6,23]_ = 6.41, *p* < 0.01). *Post hoc* analysis showed that the angle was significantly higher in block 6 than block 2 (*p* < 0.01) and block 3(*p* = 0.01); higher in block 5 than block 3 (*p* < 0.01) and block 2 (*p* < 0.01). There was a significant trial effect in the control (*F*_[6,21]_ = 3.15, *p* = 0.02) and auditory (*F*_[6,353]_ = 4.35, *p* < 0.01) conditions for peak ankle flexion angle. In the auditory condition, block 6 was higher than block 4 (*p* = 0.02) and block 1 (*p* = 0.02); block 3 was higher than block 4 (*p* < 0.01) and block 1 (*p* < 0.01).

#### Influence of Initial Spine Posture

**Figure 6:**
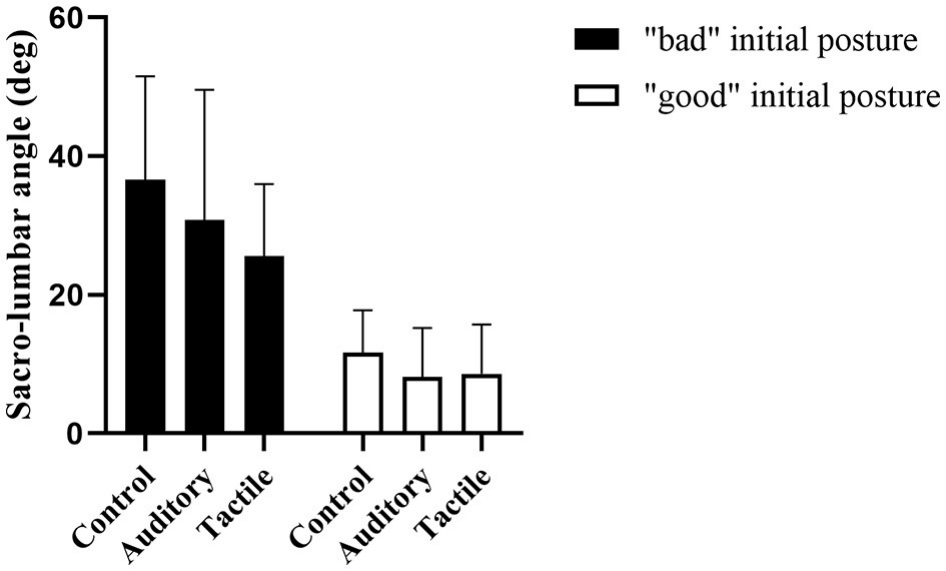
Condition effect in initially “good” and “bad” spine posture to visually show the interaction between initial movement capabilities and feedback modality. The two groups were defined using a median split on the baseline performance at 20.5°.

Results showed a statistically significant effect of condition (*F*_[1,36]_ = 6.50, *p* = 0.015), baseline (*F*_[1,36]_ = 43.52, *p* < 0.01) and condition*baseline (F_[1,36]_ = 24.61, *p* < 0.01). When a standard deviation was added to baseline, condition effect was statistically significant (*F*_[1,18]_ = 12.5, *p* < 0.01) and the estimate of condition effect was 12.4, while condition was not significant when a standard deviation was subtracted to baseline (*p* = 0.9) and the estimate was 0.26. Figure 6 graphically shows this effect with a median split of baseline performance.

### Discussion of the Pilot Study

This pilot study compared the effectiveness of auditory and tactile feedback in reducing lumbar spine flexion in a repetitive lifting task. As hypothesized, both feedback conditions promoted a reduction of lumbar spine flexion relative to a no-feedback control condition, and tactile was more effective than auditory (Table 2; Figure 4A). Furthermore, confirming our second hypothesis, this flexion reduction was maintained in both feedback conditions in retention trials without feedback, suggesting that learning was starting to occur (Figure 4B). Interestingly, the spine flexion reduction was achieved differently in the two feedback conditions. While knee flexion increased in both conditions, participants increased flexion of the hip and reduced flexion of the lower trunk in tactile, while they increased extension of the upper trunk in the auditory (Figure 3). In other words, participants tilted their hip forward and reduced flexion of the lower spine in tactile, and they primarily extended the upper spine in the auditory. This indicates that feedback modality influenced how trunk flexion was redistributed along the upper spine and lower limbs.

We also hypothesized a higher reduction of performance (i.e., increase in lumbar spine flexion) from acquisition to retention trials in the tactile than auditory condition. Lumbar spine angle increased across trials in tactile while it did not in auditory, confirming this hypothesis (Figure 5). It is however quite difficult to interpret whether these results indicate a guidance effect of the tape or some other mechanism. Lumbar spine flexion did not change from block 6 to retention, as it would typically happen with a guidance effect. However, spine flexion in retention was higher than blocks 1, 2, 3, and a similar increase was observed in blocks 4, 5, and 6 (Figure 5). This trend does not correspond to a typical learning effect of augmented feedback, whereby performance improves across trials. Two potential mechanisms can explain this: a guidance effect occurred, or the high improvement in blocks 1, 2, and 3 were physically demanding and difficult to maintain for more than 15 consecutive lifts at a relatively fast pace. Considering that the tape peeled off in most participants throughout the acquisition phase, this performance reduction from block 4 may have resulted from a lack of feedback from the tape, potentially supporting the guidance hypothesis explanation.

We lastly hypothesized a different effect of feedback modality on spine reduction depending on participants' habitual (i.e., in control condition) spine posture during lifting, with bad habitual posture (high spine flexion) benefitting more from tactile than auditory feedback. First of all, it is important to highlight that the high standard deviation in the descriptive statistics (Figures 3, 4) indicate a high variability across participants in spine control and movement kinematics. Participants responded to feedback and changed movement differently. Furthermore, participants with a habitually more-flexed lumbar spine improved more in tactile than auditory, while participants with less-flexed lumbar spine improved equally with the two feedback modalities, confirming our hypothesis (Figure 6).

The results of this study support previous research on the positive effect of providing augmented feedback on lumbar spine angle for reducing its flexion in lifting (Kernozek et al., 2006; Lavender et al., 2007; Matheve et al., 2018; Pinto et al., 2018; Boocock et al., 2019; Lorenzoni et al., 2019; Punt et al., 2020). This is the first study comparing the effect of different modalities of presenting lumbar spine angle—tactile and auditory feedback. Both mapping lumbar spine angle to a sound frequency (i.e., movement sonification), and applying a sport leukotape onto lumbar extensor muscles reduced lumbar spine flexion. These results confirm that movement sonification is a suitable strategy for enhancing control of postural behavior (Dozza et al., 2011; Lorenzoni et al., 2019). It also confirms that the simple strategy of applying tape to the low back area is effective in reducing spine flexion (Pinto et al., 2018). There was some indication that tactile feedback elicited a higher flexion reduction than auditory.

The two feedback modalities had a different effect on how spine flexion was controlled. A limited number of studies have previously examined how lumbar flexion is redistributed along upper trunk segments and lower limbs during lifting. The results of the current study showed that auditory feedback primarily elicited an increase in knee flexion (similar to Boocock et al., 2019; Punt et al., 2020) and an increase in upper trunk extension, while tactile feedback promoted participants increasing knee flexion and also increasing hip flexion (similar to Pinto et al., 2018) and reducing lower trunk flexion (Figure 3).

The effect of feedback modality interacted with participants' habitual spine flexion during lifting, and participants benefitted from the two feedback modalities depending on their tendency to lift with a more or less flexed lumbar spine. Participants that already lifted with a relatively low spine flexion in the control condition (“good” initial spine posture) benefitted equally from the two modalities, while participants with a high lumbar flexion during the baseline/control condition (“bad” initial spine posture) benefitted more from tactile than auditory feedback (Figure 6). While some of the participants might have simply ignored the instruction “avoid bending your back during lifting” in the control condition, we speculate that participants with a “bad” initial spine posture had a relatively low ability to perceive and control their spine posture. As such, they might have encountered challenges in mapping the augmented sound to their movement (Sigrist et al., 2013a). Anecdotally, some of the participants with “bad” initial posture told the experimenter at the end of the session that the sound was frustrating because they did not know how to change their movement to reduce sound frequency. On the opposite, the tape provided information directly on the area of interest and was easier for them to interpret and use the feedback. Similarly, previous research has shown that novices (with low initial performance) benefitted more from haptic feedback than movement sonification in a rowing-type movement (Sigrist et al., 2013b). This interaction between initial movement ability/tendency and feedback effect and the variability in movement kinematics across participants confirm that different individuals adopt different movement strategies to control spine flexion. Furthermore, it shows that augmenting information about spine flexion is a suitable strategy for encouraging participants to find and use their own functional strategy to reduce spine flexion, as opposed to constraining individuals into a predetermined ideal technique. We encourage future research to investigate this important issue more thoroughly. For example, another possible explanation is that the tape reduced spine flexion by limiting movement, and not by facilitating the mapping of feedback onto movement.

While tactile feedback was more effective than auditory feedback for controlling the spine, there is some indication that auditory might have been more beneficial for learning. In retention trials, lumbar spine flexion was lower in both feedback modalities than control, and there was only a trivial (*d* = 0.10) difference between the two feedback modalities (Figure 4). This trend seems to indicate that learning occurred in both conditions. However, in the tactile condition, performance decreased across trials and it was significantly lower in retention than first trial blocks (Figure 5B). This seems to suggest that tactile feedback acted as guidance and promoted the large performance improvement in the first trial blocks, but this effect vanished as the tape peeled off in the last trial blocks and was removed in the retention block. This guidance effect was also observed in previous research (Sigrist et al., 2013b). Contrarily, this effect was not observed in the auditory condition. These results confirm that movement sonification is a suitable feedback strategy to promote learning (Dyer et al., 2017; Hasegawa et al., 2017; Ghai et al., 2019), while tactile feedback primarily promotes performance and performance deteriorates when feedback is removed (Sigrist et al., 2013a).

This study presents some limitations that should be considered when interpreting the results. It was a pilot study with limited statistical power and experimental design (i.e., within-subject) for examining in depth the learning effect of feedback modalities. Furthermore, we recruited university students without any experience in lifting in the workplace (e.g., material handling), and the picture might be a bit different in workers. Therefore, the results should be primarily considered in the broad context of this article to inform future research design and not to generalize the observed behavior to the working population. Furthermore, the rigid tape used in the tactile condition was not reliably attached to participants' back throughout the practice trials, and peeled off at different rates across participants. This likely produced noise in the data of the tactile condition, and prevented from evaluating whether the guidance effect occurred in this condition. Future research is encouraged to examine how tapes of varying stiffness can concurrently be stiff enough to elicit a tactile response and flexible enough to adhere to the skin in spine-flexed postures. Lastly, the short duration of the retention period (only 6 min after practice) limits our evaluation of any learning effect of feedback, and we suggest future research to include retention tests of longer duration.

In summary, both feedback modalities are beneficial for reducing lumbar spine flexion, but it has to be confirmed whether the reduction is retained in the long-term when feedback is removed (current and previous research has only used short-term retention). Particularly, this should be investigated in the tactile feedback, whereby guidance is likely to occur. The tape can represent an easy-to-implement and economically sustainable strategy for the occupational sector, and it should be examined whether it can be used to assist only or also to train workers. Furthermore, this study suggests future research to assess and account for the variability in participants' response to augmented feedback and for the influence of initial movement ability/tendency on feedback modality effectiveness. This study provides additional indication that an optimal technique that fits all individuals does not exist, and a training intervention should adopt strategies that promote each worker finding their own functional and safe movement coordination. This includes exposing workers to different feedback modalities and evaluate which modality works best for them. Perhaps, a “smart” tape that can also provide auditory feedback could be a suitable option for delivering both tactile and auditory feedback.

## General Discussion and Conclusion

In this article, we have argued for a change in the way safe lifting is trained in the occupational domain, moving from the traditional educational approach to implementing the deliberate practice framework directly in the workplace. This approach will embody knowledge on safe movement and embed deliberate practice in the workplace, promoting workers' development of their *own* knowledge and execution of their *own* safe movement (perception, cognition, and action) functional to the environment they work in. Psychology, which has been typically overlooked in the process, will gain a relevant role in creating the right conditions for promoting deliberate practice in the workplace with the goal of improving one's own lifting behavior. Augmented feedback holds the key for achieving such goal, as it will create suitable training conditions whilst allowing a worker to perform work duties as they normally would. Principles from motor skill learning literature can inform the design of an effective feedback strategy, and lab-based research can provide benefits and constraints of different feedback options.

While the discussed approach can be used to target different movement-related risk factors, we targeted spine flexion. Flexion of the lumbar area during repetitive lifting is a prevalent risk factor for low back injuries and previous research has shown that providing feedback on lumbar spine is effective in reducing peak lumbar flexion during lifting. According to key requirements for deliberate practice conditions, feedback has to be informative, individualized, and actionable. Feedback modality is a critical component to ensure workers can perceive and regulate their action on the content delivered (i.e., feedback is actionable). We have conducted a pilot study to examine the benefits and boundary conditions of two modalities (auditory and tactile) of providing feedback on spine flexion in a simulated repetitive lifting task. The main findings can be summarized as follow: (i) quality of practice is critical for the effectiveness of an intervention—mere practice (control condition) did not reduce spine flexion while both feedback modalities reduced it, (ii) the two feedback modalities promoted lifting performance and learning differently—tactile feedback primarily reduced spine flexion in practice (i.e., guidance effect) while auditory feedback showed sign of learning, (iii) participants' initial lifting performance interacted with feedback modality—low initial performers benefitted more from tactile than auditory feedback, while high initial performers benefitted equally from the two feedback modalities, and iv) the two feedback modalities promoted spine flexion reduction through different changes in movement—changes in hip and lower trunk in tactile feedback while changes in upper trunk in auditory.

The results of this pilot study, combined with existing lab-based evidence, can provide initial insights on benefits and constraints of augmented feedback for implementing deliberate practice in the workplace. Mere practice does not improve lifting; augmented feedback on spine flexion can make practice effortful, individualized, goal-oriented and (more or less) guided (key requirements for deliberate practice), in turn improving lifting performance; different feedback modalities can convey informative and individualized information, but depending on a worker's lifting ability or current movement tendency one modality may be more “actionable” than another modality (e.g., tactile is more “actionable” than auditory in novices); different modalities may be more effective for enhancing lifting performance or promoting learning of safe lifting movement. This initial evidence suggests that feedback may indeed be critical in transforming routine, repetitive lifting duties into deliberate practice of lifting movement with the intention of improving one's own lifting behavior. This, in turn, is expected to reduce injury risks generating from incorrect spine posture during lifting.

We acknowledge that implementing such strategy in the workplace will be challenging, and a positive transfer from laboratory to occupational context is not straightforward. Outside of the controlled laboratory environment, a number of factors interact and influence the effectiveness of a strategy. For instance, simply providing a custom auditory feedback on spine flexion has been shown to be ineffective in improving spine posture in workers (Ribeiro et al., 2020). As previously discussed, feedback interact with individual and situational factors and a feedback strategy has to cater for these factors to be effective (see Oppici et al., 2021). In this context, pilot studies conducted in the laboratory are essential for providing the benefits and conditions of feedback for reducing dangerous lifting behavior. The research conducted and examined in this study represents an example of this approach, and provides a first step in delineating the key features of a suitable feedback strategy. Future research should then investigate the interaction of such feedback strategy with other learning factors and how it can be effectively implemented in the workplace. Furthermore, it should be investigated, with a bigger sample size, whether gender interacts with the feedback effect.

In conclusion, we have put forward our suggestion, with a detailed rationale, for changing the wide-spread but ineffective approach of teaching lifting behavior (i.e., the educational approach). We think that psychology has to gain a central role for improving current practice. Deliberate practice is effective in a variety of domains that require skilled performance of perceptual-motor and perceptual-cognitive skills, and we believe that it can highly benefit the occupational domain too. We have also shown how lab-based research can inform the implementation of feedback in a deliberate practice intervention. Certainly, the effectiveness of this approach has to be put to test, but, if anything, we hope for our article to be thought provoking and to stimulate further research in this perspective.

## Data Availability Statement

The original contributions presented in the study are included in the article/supplementary files, further inquiries can be directed to the corresponding author.

## Ethics Statement

The studies involving human participants were reviewed and approved by Ethikkommission an der TU Dresden, Technische Universität Dresden. The patients/participants provided their written informed consent to participate in this study.

## Author Contributions

LO and SN: conceptualization. LO, KG, and SN: study design. LO, KG, AG, and RR: data collection and data analysis. LO, KG, AG, RR, and SN: writing and reviewing the manuscript. All authors contributed to the article and approved the submitted version.

## Funding

This work was funded by the German Research Foundation (DFG, Deutsche Forschungsgemeinschaft) as part of Germany's Excellence Strategy—EXC 2050/1—Project ID 390696704—Cluster of Excellence Centre for Tactile Internet with Human-in-the-Loop (CeTI) of Technische Universität Dresden. The funding source had no involvement in this article.

## Conflict of Interest

The authors declare that the research was conducted in the absence of any commercial or financial relationships that could be construed as a potential conflict of interest.

## Publisher's Note

All claims expressed in this article are solely those of the authors and do not necessarily represent those of their affiliated organizations, or those of the publisher, the editors and the reviewers. Any product that may be evaluated in this article, or claim that may be made by its manufacturer, is not guaranteed or endorsed by the publisher.
